# Melatonin mitigates oxidative damage induced by anthracycline: a systematic-review and meta-analysis of murine models

**DOI:** 10.3389/fcvm.2023.1289384

**Published:** 2023-11-23

**Authors:** Andrea Faggiano, Elisa Gherbesi, Ashot Avagimyan, Massimiliano Ruscica, Luca Donisi, Maria Antonia Fedele, Carlo Maria Cipolla, Marco Vicenzi, Stefano Carugo, Daniela Cardinale

**Affiliations:** ^1^ Department of Cardio-Thoracic-Vascular Diseases, Foundation IRCCS Ca’ Granda Ospedale Maggiore Policlinico, Milan, Italy; ^2^ Department of Clinical Sciences and Community Health, University of Milan, Milan, Italy; ^3^ Department of Anatomical Pathology and Clinical Morphology, Yerevan State Medical University after M. Heratsi, Yerevan, Armenia; ^4^ Department of Pharmacological and Biomolecular Sciences “Rodolfo Paoletti”, University of Milan, Milan, Italy; ^5^ Cardioncology Unit, Cardioncology and Second Opinion Division, European Institute of Oncology, I.R.C.C.S., Milan, Italy

**Keywords:** melatonin, anthracycline, cardiotoxicity, oxidative stress, oxidative damage

## Abstract

**Background:**

Oxidative stress induced by the excessive production of reactive oxygen species is one of the primary mechanisms implicated in anthracycline (ANT)-induced cardiotoxicity. There is a strong clinical need for a molecule capable of effectively preventing and reducing the oxidative damage caused by ANT. In vitro and *in vivo* studies conducted in mice have shown that melatonin stimulates the expression of antioxidative agents and reduces lipid peroxidation induced by ANT.

**Methods:**

We investigated this issue through a meta-analysis of murine model studies. The outcome of the meta-analysis was to compare oxidative damage, estimated by products of lipid peroxidation (MDA = Malondialdehyde) and markers of oxidative stress (SOD = Superoxide Dismutase, GSH = Glutathione), along with a marker of cardiac damage (CK-MB = creatine kinase–myocardial band), assessed by measurements in heart and/or blood samples in mice undergoing ANT chemotherapy and assuming melatonin vs. controls. The PubMed, OVID-MEDLINE and Cochrane library databases were analysed to search English-language review papers published from the inception up to August 1st, 2023. Studies were identified by using Me-SH terms and crossing the following terms: “melatonin”, “oxidative stress”, “lipid peroxidation”, “anthracycline”, “cardiotoxicity”.

**Results:**

The metanalysis included 153 mice administered melatonin before, during or immediately after ANT and 153 controls from 13 studies. Compared with controls, the levels of all oxidative stress markers were significantly better in the pooled melatonin group, with standardized mean differences (SMD) for MDA, GSH and SOD being −8.03 ± 1.2 (CI: −10.43/−5.64, *p* < 0.001), 7.95 ± 1.8 (CI: 4.41/11.5, *p* < 0.001) and 3.94 ± 1.6 (CI: 0.77/7.12, *p* = 0.015) respectively. Similarly, compared with controls, CK-MB levels reflecting myocardial damage were significantly lower in the pooled melatonin group, with an SMD of −4.90 ± 0.5 (CI: −5.82/−3.98, *p* < 0.001).

**Conclusion:**

Melatonin mitigates the oxidative damage induced by ANT in mouse model. High-quality human clinical studies are needed to further evaluate the use of melatonin as a preventative/treatment strategy for ANT-induced cardiotoxicity.

## 1. Introduction

Anthracyclines (ANT) are very effective drugs for the treatment of different types of malignant cancers. However, ANT clinical application is dose-limited due to the potential side effect of cardiotoxicity, in particular for doxorubicin (DOX) (1).

Multiple mechanisms involved in ANT-induced cardiotoxicity have been explored and the increased oxidative stress by the overproduction of reactive oxygen species (ROS) is one of the main hypothesis suggested in literature (2). Cardiomyocytes are enriched in mitochondria, a major subcellular target of ANT, due to its affinity to cardiolipin, a component of the inner mitochondrial membrane (3). The heart is vulnerable to ANT-induced oxidative damage because of the abundancy of mitochondria and the relatively low levels of antioxidant enzymes compared to other tissues,. As a result, a reduction of oxidative stress can be considered a clinical target for ANT-induce cardiotoxicity prevention and/or treatment (3).

Melatonin (N-acetyl-5-methoxytryptamine), a secretory product of the pineal gland, is a ROS scavenger of high potency, and with its amphiphilic property, melatonin is capable of crossing mitochondrial membrane (4, 5). *In vitro* and *in vivo* studies (e.g., in mice) reported that melatonin stimulates of the expression of antioxidative agents including Superoxide Dismutase (SOD) and Glutathione (GSH) and reduces lipid peroxidation induced by ANT, estimated by the dosage of Malondialdehyde (MDA), product of this process (6–32).

Evidence from clinical studies in humans evaluating the specific effect of melatonin in the prevention and/or treatment of ANT-induced cardiotoxicity are lacking. Only a study is currently available, which is focused on patients with advanced-stage solid tumors and poor functional status and found that melatonin reduced the occurrence of cardiotoxicity (33). Our hypothesis is that melatonin may prove effective in preventing and counteracting the oxidative damage induced by ANT, and thus play a role in preventing cardiotoxicity.

Therefore, having supportive basic science data on the role of melatonin in this setting from single studies on mouse model, we investigated this issue through a meta-analysis of studies that reported data on molecular oxidative damage in mice undergoing ANT chemotherapy and supplemented with melatonin vs. controls.

## 2. Methods

The present research was performed following the Preferred Reporting Items for Systematic reviews and Meta-Analyses (PRISMA) guidelines (34). The PubMed, OVID-MEDLINE and Cochrane library databases were analysed to search English-language review papers published from the inception up to August 1st, 2023. Studies were identified by using Me-SH terms and crossing the following terms: “melatonin”, “oxidative stress”, “lipid peroxidation”, “anthracycline”, “cardiotoxicity”. Main inclusion criteria were: (1) English papers published in peer-reviewed journals; (2) studies providing parameters of lipid peroxidation, oxidative stress, and/or cardiac damage; (3) minimum set of mouse model population characteristics. Specific exclusion criteria were: (1) studies with less than 4 mice; no predetermined maximum sample size was set; (2) studies reporting data on oxidative damage with incompatible and/or non-convertible units of measurement and therefore not amenable to metanalysis; (3) reviews, editorials, and case reports were excluded from analyses (but examined for potential additional references).

Literature search and data extraction were performed by two reviewers (A.F. and L.D) and independently checked by another reviewer (E.G) that resolved disagreements on study judgments. Where complete data were not available, they were requested from the specific corresponding author and, if it was not possible to obtain them, they were extrapolated from the figures of the studies. Although it has not been specifically designed for basic science research studies, the Newcastle– Ottawa Scale (NOS) was used to measure study quality (http://www.ohri.ca/programs/clinical_epidemiology/oxford.htm).

The outcome of the meta-analysis was to compare oxidative damage estimated by products of lipid peroxidation and markers of oxidative stress (ie, MDA, GSH, SOD), along with marker of cardiac damage (creatine kinase–myocardial band CK-MB), assessed by dosage in heart and/or blood samples, in mice undergoing ANT chemotherapy and assuming melatonin (before, during, or after chemotherapy) vs. controls not assuming melatonin. To this purpose, a pooled analysis of these parameters was performed using fixed or random effects models by Comprehensive Meta-Analysis Version 2, Biostat, Englewood, NJ, USA. Heterogeneity was estimated by using I-square, Q and tau-square values; random or fixed effect models were applied when heterogeneity across studies (*I*^2^) was higher or lower than 75%, respectively. Standard means difference (SMD) with 95% confidence interval (CI) was calculated to evaluate the statistical difference of variables in melatonin treated mice and controls. The limit of statistical significance was set at *P* < .05. Publication bias was assessed by using the funnel plot method according to the trim and fill test. Observed and adjusted values, their lower and upper limits have been calculated. To assess the effect of individual studies on the pooled result, we conducted a sensitivity analysis by excluding each study one by one and recalculating the combined estimates on remaining studies.

## 3. Limitations

Our meta-analysis has several limitations that we would like to address. Some of the variables evaluated in the included studies are heterogeneous and expressed in different units of measurement, requiring conversion. Certain studies were excluded precisely due to the inability to render the data amenable to meta-analysis. Additionally, despite all the various regimens of ANT chemotherapy administered in the studies being sufficient to induce cardiotoxicity, they vary greatly from one another, even in terms of time frames (e.g., models of acute, subacute, chronic toxicity). Similarly, the administration of melatonin is not consistent across the different studies in terms of dosage, route and timing of administration. Furthermore, as none of the studies evaluated the potential gender-specific efficacy of melatonin, no conclusions can be drawn regarding its gender-related impact in this setting. Finally, our meta-analysis focused solely on those outcomes related to oxidative damage that were amenable for meta-analysis, while others exist in the literature (e.g., nitric oxide, glutathione peroxidase enzyme activity, 4-hydroxyalkenals, thiobarbituric acid reactive substances, conjugated dienes, and protein carbonyl) but lack sufficient data for a meta-analytic study.

## 4. Results

### 4.1. Characteristics of the studies

**Figure 1:**
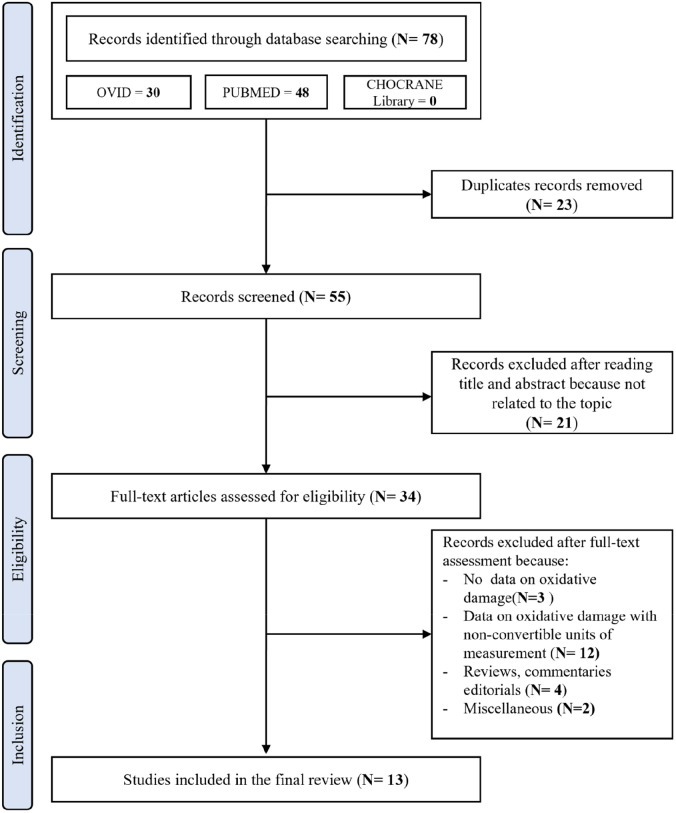
PRISMA flowchart showing the search strategy and manuscripts selection process.

After removing duplicates, the initial literature search identified 55 papers. The PRISMA flowchart showing the search strategy and manuscripts selection process is illustrated in Figure 1.

After the initial screening of titles and abstracts, 21 studies were excluded as they were not related to the topic. Therefore, 34 studies were reviewed; of these, 3 did not report data on oxidative damage, 11 reported data on oxidative damage with incompatible and/or non-convertible units of measurement and therefore not amenable to metanalysis, 4 were reviews, commentaries, and editorials, and 2 were excluded for miscellaneous reasons. According to the NOS, the quality of the studies ranged from 6 to 9 (i.e., a score that identifies studies of fair or good quality). Therefore, no study was excluded based on its limited quality.

On the whole 306 mice undergoing ANT chemotherapy were included in 13 studies (sample size ranging from 12 to 70) performed in 3 continental areas (Europe = 1, Asia = 10, Africa = 2): 153 mice assuming melatonin before, during or immediately after chemotherapy and 153 controls not assuming melatonin (6–16, 18, 19). Since the study of Oz et al. (10) included 3 different analysable groups (melatonin before ANT group called “OZ pre”, melatonin after ANT group called “Oz post”, and control group) it was considered twice. For this reason, despite the actual number of included studies being 13, the meta-analysis was conducted on 14 different melatonin treatment groups vs. control groups. Table 1 summarizes the main findings of selected studies such as Authors, year of publication, sample size, chemotherapy regimen, timing and route of melatonin administration, mice species, gender and biomarkers of oxidative damage (MDA, GSH, SOD and CK-MB) assessed at the end of the chemotherapy and melatonin cycles. The cumulative dose (mg/kg) of ANT which was sufficient to develop cardiotoxicity was administered to each mouse, generally intraperitoneally (11 intraperitoneal and 3 intravenous). The comparison of biomarkers of oxidative stress between mice assuming melatonin and controls was conducted at the end of the chemotherapeutic and melatonin cycles (chemotherapy duration ranging from 1 to 30 days, melatonin duration ranging from 3 to 30 days). Notably, only one study included female mice, the adopted ANT was DOX in every study, the preferred melatonin route of administration was intraperitoneal (10 studies intraperitoneal, 2 studies subcutaneous, 2 studies oral) and melatonin administration mainly started before the chemotherapy regimen (5 studies before + during + immediately after, 4 studies during, 3 studies only before, 2 studies during + immediately after, 1 study only immediately after chemotherapy).

**Table 1:** Summary of study characteristics of oxidative damage biomarkers in mice included in the systematic review and meta-analysis.

Author, Publication year	ANT drug name	ANT scheme of administration	MEL scheme of administration	Timing of MEL administration	Mice Species	Male (%)	Sample size (*N*)		Heart MDA levels (nmol/mg)		Heart GSH levels (*μ*mol/g)		Blood SOD levels (U/mg)		Blood CK-MB levels (U/l)	
							MEL	Controls	MEL	Controls	MEL	Controls	MEL	Controls	MEL	Controls
Wahab et al. (6)	DOX	4 mg/kg/week for 30 days, IP	5 mg/kg/day for 30 days, Oral	During ANT	Swiss Albino	100	30	40	125 ± 10	180 ± 12	85 ± 2	65 ± 2	N.A.	N.A.	N.A.	N.A.
Dziegel et al. (7)	DOX	2.5 mg/kg/week for 30 days, IP	20 mg/kg/week for 30 days, SC	During ANT	Buffalo Strain	10	10	10	N.A.	N.A.	N.A.	N.A.	780 ± 200	760 ± 300	N.A.	N.A.
Sahna et al. (8)	DOX	20 mg/kg/day for 1 day, IP	4 mg/kg/day for 3 days, IP	During (1 day) + after ANT (2 days)	Wistar Rats	0	8	8	43 ± 2	58 ± 2	N.A.	N.A.	N.A.	N.A.	N.A.	N.A.
Balli et al. (9)	DOX	3 mg/kg/day for 4 days, IP	6 mg/kg/day for 15 days, IP	During (4 days) + after ANT (11 days)	Wistar Rats	100	8	8	6 ± 0.1	14 ± 4	N.A.	N.A.	N.A.	N.A.	N.A.	N.A.
Oz et al. (10)	DOX	45 mg/kg/day for 1 day, IV	10 mg/kg/day for 7 days, SC	Before ANT	Wistar Rats	100	10	10	85 ± 9	160 ± 117	3.8 ± 0.4	2.9 ± 0.3	N.A.	N.A.	519 ± 35	803 ± 80
				After ANT			10		70 ± 4		5.4 ± 0.4		N.A.		275 ± 15	
Othman et al. (11)	DOX	10 mg/kg/day for 1 day, IP	15 mg/kg/day for 10 days, IP	Before (4 days) + during (1 day) + after ANT (5 days)	Sprague Dawley	100	10	10	N.A.	N.A.	5.2 ± 0.2	2.3 ± 0.1	N.A.	N.A.	N.A.	N.A.
Aydemir et al. (12)	DOX	15 mg/kg/day for 1 day, IP	5 mg/kg/day for 9 days, IP	Before (2 days) + during (1 day) + after ANT (6 days)	Wistar Rats	N.A.	8	8	N.A.	N.A.	N.A.	N.A.	1,200 ± 30	980 ± 20	N.A.	N.A.
Ozturk et al. (13)	DOX	20 mg/kg/day for 1 day, IP	20 mg/kg/days for 5 days, Oral	Before (1 days) + during (1 day) + after ANT (3 days)	Sprague Dawley	100	8	6	N.A.	N.A.	N.A.	N.A.	1,100 ± 80	760 ± 20	150 ± 50	450 ± 50
Alghasham et al. (14)	DOX	2.5 mg/kg/day for 5 days, IP	5 mg/mg/days for 5 days, IP	Before ANT	Swiss Albino	100	6	6	N.A.	N.A.	N.A.	N.A.	N.A.	N.A.	240 ± 5	325 ± 19
Bilginoğlu et al. (15)	DOX	18 mg/kg/day for 3 days, IP	10 mg/kg/day for 7 days, IP	Before ANT	Wistar Albino	100	7	7	N.A.	N.A.	N.A.	N.A.	23 ± 3	15 ± 3	524 ± 90	794 ± 83
Liu et al. (16)	DOX	10 mg/kg/day for 2 days, IP	20 mg/kg/day for 7 days, IP	During ANT	C57BL/6	100	20	20	300 ± 10	600 ± 20	N.A.	N.A.	N.A.	N.A.	N.A.	N.A.
Durdagi et al. (18)	DOX	45 mg/kg/day for 1 day, IV	10 mg/kg/day for 7 days, IP	Before (4 days) + during (1 day) + after ANT (2 days)	Wistar Albino	100	6	8	200 ± 5	375 ± 15	N.A.	N.A.	N.A.	N.A.	N.A.	N.A.
Sun et al. (19)	DOX	5 mg/kg/week for 30 days, IP	10 mg/kg/week for 30 days, IP	During ANT	Sprague Dawley	100	12	12	8 ± 1	12 ± 1	4.5 ± 0.2	3.8 ± 0.1	N.A.	N.A.	N.A.	N.A.

ANT, anthracycline; DOX, doxorubicin; MEL, melatonin; MDA, Malondialdehyde; GSH, glutathione; SOD, superoxide dismutase; CK-MB, creatine kinase–myocardial band isoenzyme; IP, intraperitoneally; IV, intravenous; SC, subcutaneously; N.A, not available

### 4.2. Effect of melatonin on heart MDA levels

**Figure 2:**
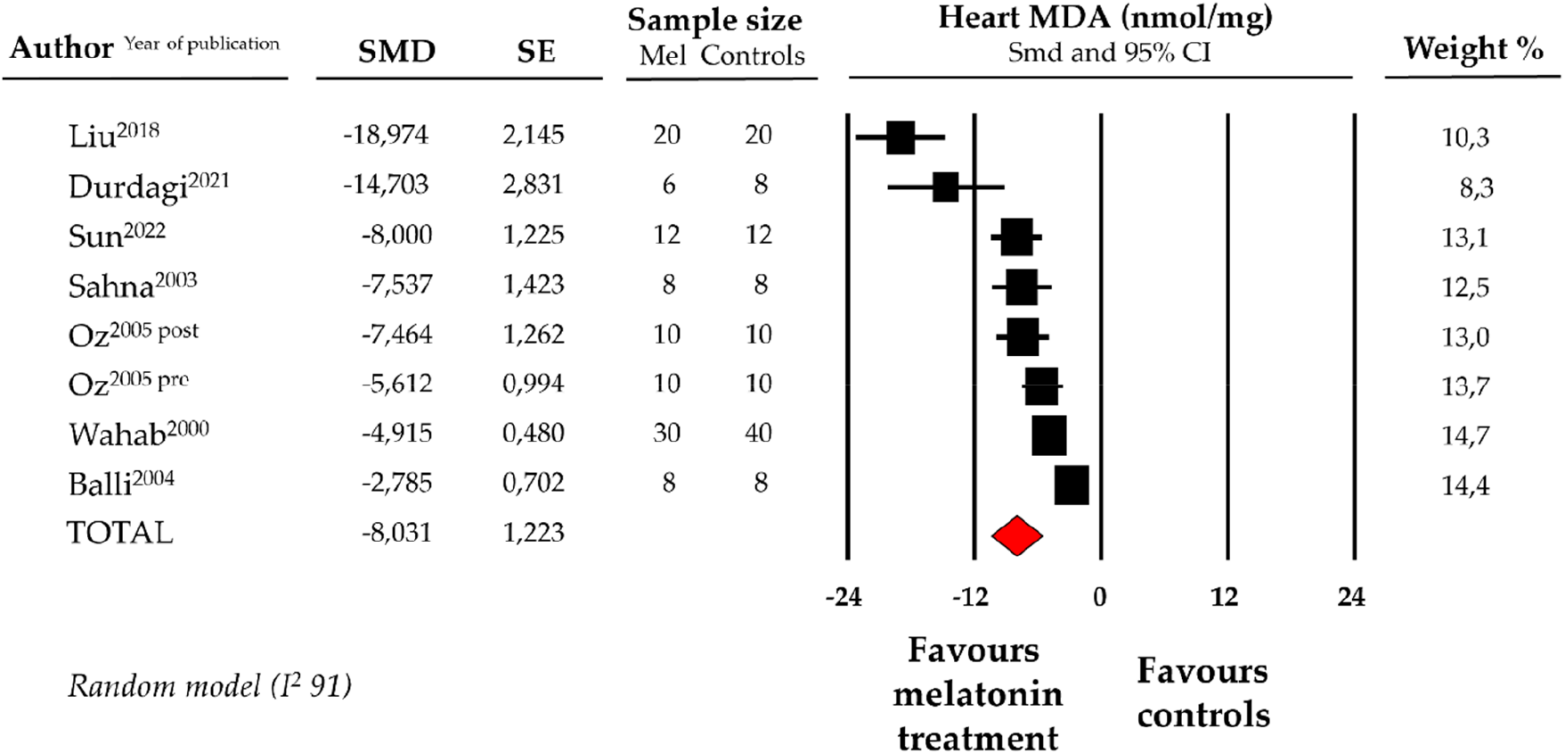
Forest plot for standard means difference (SMD) of heart malondialdehyde (MDA) levels in melatonin treated mice and controls. Relative weight of each study is reported on the right side. CI, confidence interval.

Compared with controls (*N* = 106), MDA levels measured on heart tissue samples after ANT chemotherapy with DOX was significantly better in the pooled melatonin group (*N* = 104). As shown in Figure 2, MDA levels (104.2 ± 6.8 vs. 194.5 ± 26.7 nmol/mg, data from 7 studies and from 8 vs. 8 analysable groups) were significantly lower in the pooled melatonin group than in the control group; SMD being −8.03 ± 1.2 (CI: −10.43/−5.64, *p* < 0.001). The presence of a single study effect was excluded at sensitivity analysis; a relevant publication bias was not present for studies reporting MDA in melatonin mice and controls. The difference in MDA levels between the melatonin group and controls was still present after correction for publication bias (SMD: −9.19, CI: −12.56/−5.82).

### 4.3. Effect of melatonin on heart GSH levels

**Figure 3:**
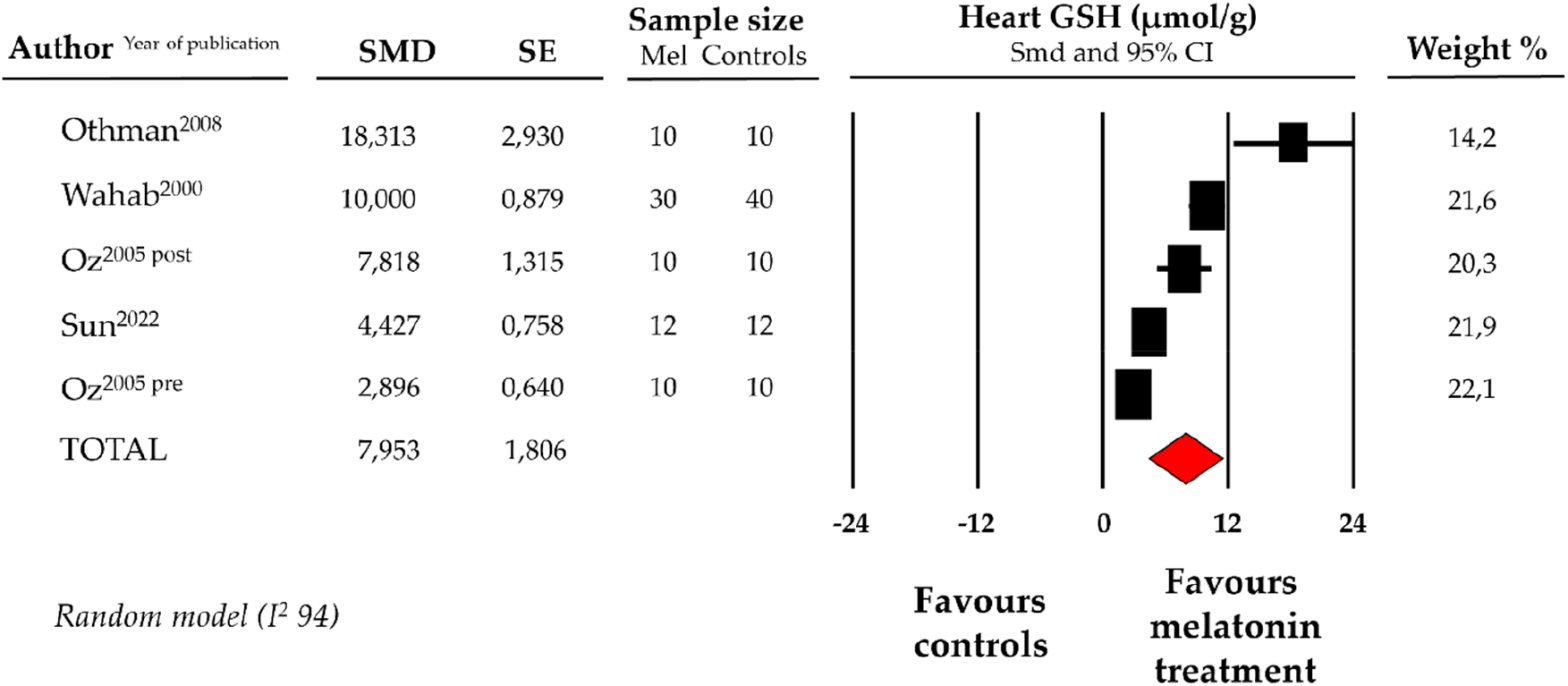
Forest plot for standard means difference (SMD) of heart glutathione (GSH) levels in melatonin treated mice and controls. Relative weight of each study is reported on the right side. CI, confidence interval.

Compared with controls (*N* = 72), GSH levels measured on heart tissue samples after ANT chemotherapy with DOX were significantly better in the pooled melatonin group (*N* = 72). As shown in Figure 3, GSH levels (20.8 ± 4.5 vs. 15.3 ± 2.5 micromol/g, data from 4 studies and from 5 vs. 5 analysable groups) were significantly higher in the pooled melatonin group vs. control group; SMD being 7.95 ± 1.8 (CI: 4.41/11.5, *p* < 0.001). Publication bias was not present for studies reporting GSH in melatonin mice and controls.

### 4.4. Effect of melatonin on blood levels of SOD

**Figure 4:**
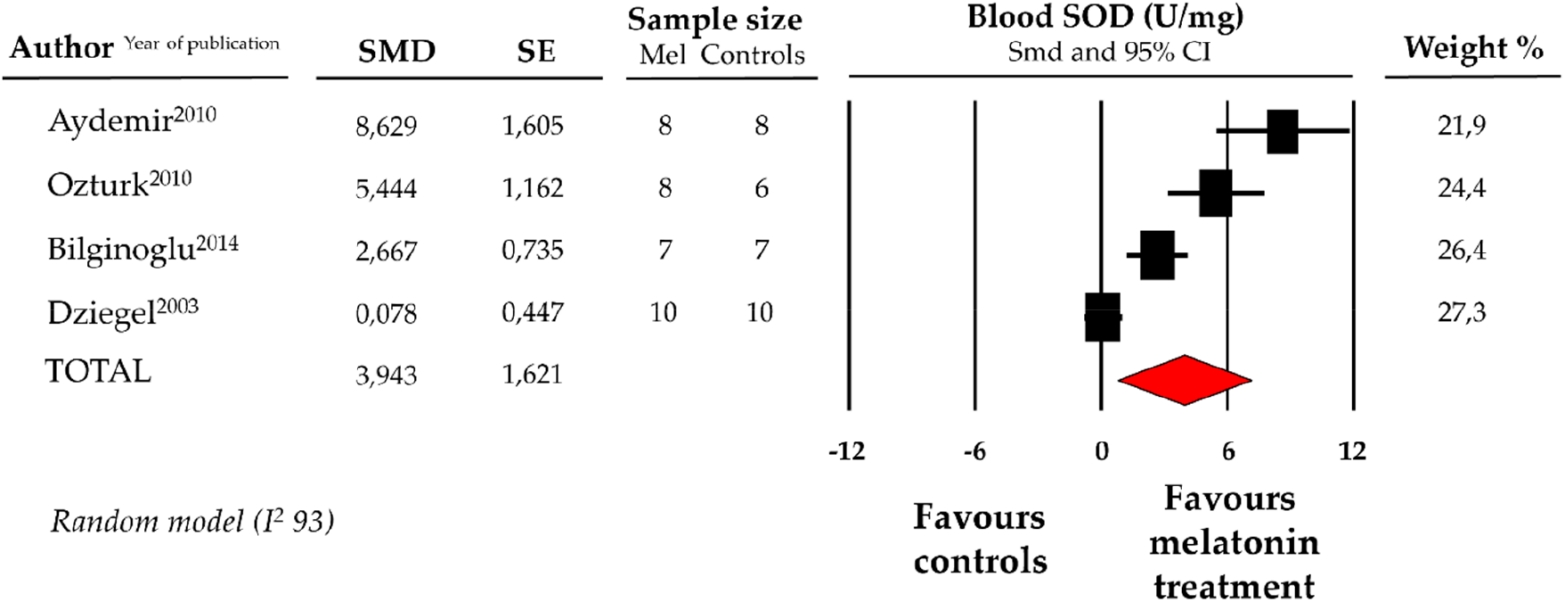
Forest plot for standard means difference (SMD) of blood superoxide dismutase (SOD) levels in melatonin treated mice and controls. Relative weight of each study is reported on the right side. CI, confidence interval.

Compared with controls (*N* = 31), SOD levels, measured on peripheric blood sample after ANT chemotherapy with DOX, were significantly better in the pooled melatonin group (*N* = 33). As shown in Figure 4, SOD levels (775.6 ± 409.1 vs. 627.97 ± 309.4 U/mg, data from 4 studies) were relevantly higher in the pooled melatonin group than in the control group; SMD being 3.94 ± 1.6 (CI: 0.77/7.12, *p* = 0.015). A difference in SOD levels between the melatonin group and controls was still present after correction for publication bias, even if not reaching statistical significance (SMD: 1.32, CI: −1.63/4.28) **(**Supplementary Figure S1).

### 4.5. Effect of melatonin on blood levels of CK-MB

**Figure 5:**
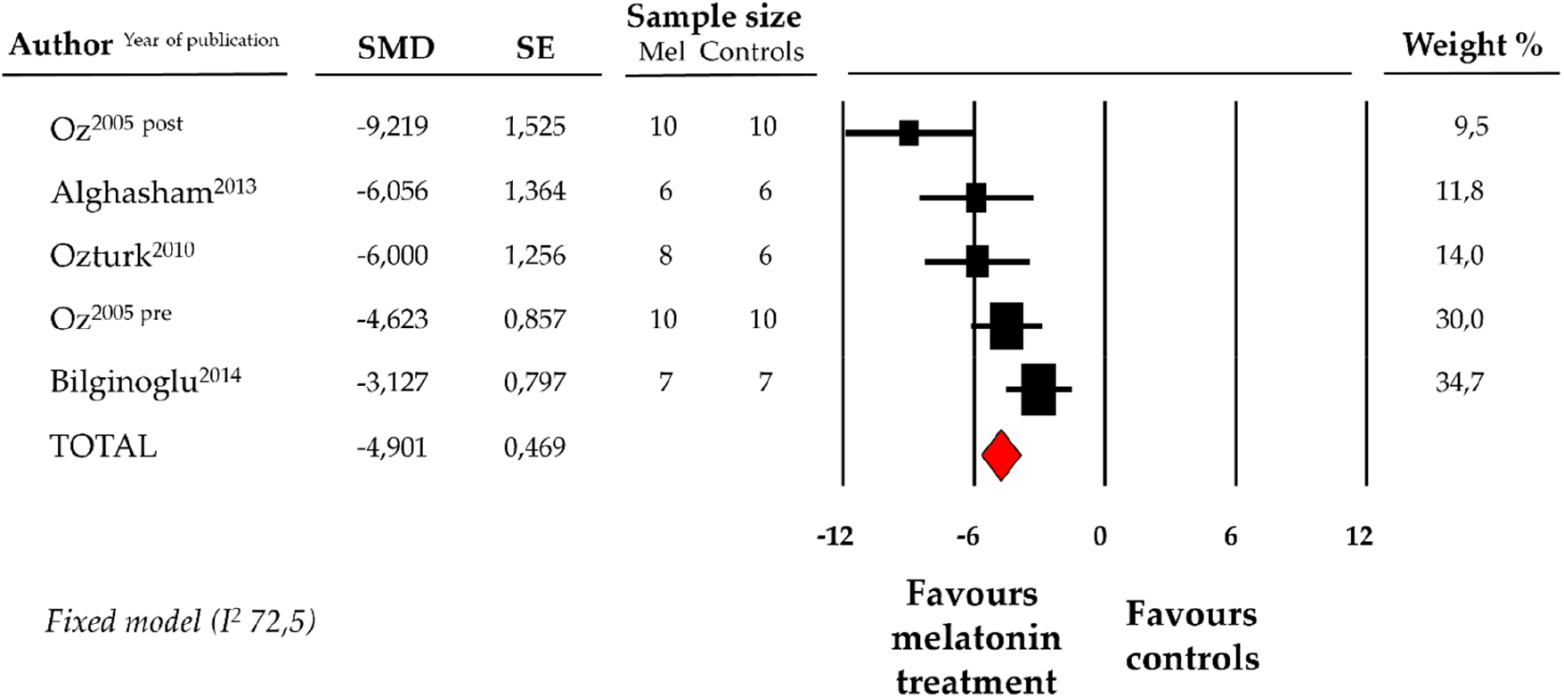
Forest plot for standard means difference (SMD) of blood creatine kinase–myocardial band (CK-MB) levels in melatonin treated mice and controls. Relative weight of each study is reported on the right side. CI, confidence interval.

Compared with controls (*N* = 29), CK-MB levels measured on peripheric blood sample after ANT chemotherapy with DOX was significantly better in the pooled melatonin group (*N* = 41). As shown in Figure 5, CK-MB levels (337.6 ± 40.2 vs. 634.5 ± 118.5 U/L, data from 4 studies and from 5 vs. 5 analysable groups) were relevantly lower in the pooled melatonin group than in the control group; SMD being −4.90 ± 0.5 (CI: −5.82/−3.98, *p* < 0.001). The presence of a single study effect was excluded at sensitivity analysis; a relevant publication bias was not present for studies reporting CK-MB in melatonin mice and controls. The difference in CK-MB levels between the melatonin group and controls was still present after correction for publication bias (SMD: −4.21, CI: −5.04/−3.37).

## 5. Discussion

Based on the existing literature, melatonin emerges as a promising cardioprotective agent in the context of ANT-induced cardiotoxicity in mouse models. Its cardioprotective effects appear to be associated not only with its ability to modulate redox system biomarkers (evaluated by MDA, GSH, and SOD levels) but also with its beneficial influence on cardiomyocyte injury, as evidenced by CK-MB levels (Table 2).

**Table 2:** Summary table of the overall effect of melatonin on oxidative damage and myocardial injury biomarkers.

Biomarker	Beneficial Effect	SMD	C.I.	*p*. value
Heart MDA	Yes	−8.03 ± 1.2	−10.43/−5.64	<0.001
Heart GSH	Yes	7.95 ± 1.8	4.41/11.5	<0.001
Blood SOD	Yes	3.94 ± 1.6	0.77/7.12	0.015
Blood CK-MB	Yes	−4.90 ± 0.5	−5.82/−3.98	<0.001

SMD, Standard Means Difference; CI, Confidence Interval; SOD, Superoxide Dismutase; GSH, Glutathione; MDA, Malondialdehyde; CK-MB, Creatine Kinase–Myocardial Band.

The myocardium is an energy-dependent tissue with a well-developed mitochondrial apparatus (35). However, the antioxidant reserve in cardiomyocytes is limited, making them highly susceptible to oxidative damage. Oxidative stress is considered one of the main mechanisms in the development and progression of many cardiovascular diseases (36). In particular, in the pathogenesis of ANT-induced cardiotoxicity, oxidative stress is recognized as a main contributor to cardiomyocytes damage and death (37). DOX, a cationic molecule with hydrophilic and hydrophobic sights, penetrates easily organelle membranes (38). Its cationic nature allows DOX to readily enter the inner mitochondrial membrane, where it forms a stable complex with cardiolipin, disrupting its function and stimulating the production of superoxide radicals responsible for oxidative damage and lipid peroxidation (39).

Currently, neurohormonal therapies and statins are the only cardiovascular drugs suggested by the European guidelines to reduce the risk of significant ANT-induced cardiotoxicity (1). Although the efficacy of statins in this setting may be in question, recent data and a 2023 meta-analysis of randomized clinical trials conducted by Agarwal et al. have shown that statins are associated with a 54% reduction in the relative risk of ANT-induced cardiac dysfunction (40, 41). In addition, sodium-glucose cotransporter 2 inhibitors have shown promising results in preventing ANT-related cardiac dysfunction on mouse models (42). Moreover, our group has recently published the first human case-series demonstrating clinical benefits of sodium-glucose cotransporter 2 inhibitors in the specific treatment of ANT-related cardiac dysfunction (43). Finally, a recent Cochrane Systematic Review and Meta-analysis demonstrated the actual efficacy of dexrazoxane in preventing and reducing cardiotoxicity in adults undergoing ANT treatment, with no evidence of a negative impact on tumor response rates (44). However, currently available medical strategies to prevent and to treat ANT-induced cardiotoxicity need further refinement. In particular, a molecule capable of preventing and/or reducing ANT-mediated oxidative damage would be highly desirable.

Within this scenario, the critical assessment of the molecular cascades of melatonin's potential cardioprotective effects and their extrapolation to the model of ANT-induced cardiotoxicity appears relevant. Molecular evidence suggests that melatonin could exert a significant cardioprotective effect, primarily through its anti-oxidant properties (45–47). Out of the numerous available antioxidants, melatonin has undergone extensive investigation for its potential to prevent ANT-induced cardiotoxicity through both *in vitro* and *in vivo* studies conducted in mice (48). Melatonin improves survival rates in mice with tumours receiving high cumulative doses of ANT and particularly benefits the structural and functional parameters of the myocardium (49). It is worth emphasizing that melatonin does not seem impairing the effectiveness of chemotherapy, positioning this hormone as a potentially useful biological compound to be used in under-treated and post-treatment cancer patients (50).

The *anti-oxidant effects* of melatonin [due to its amphiphilic nature it can easily diffuse through biological membrane and penetrate into cardiomyocytes (51) responsible for cardio-protection in experimental models of ANT-induced cardiotoxicity is hypothesized to be related to different and complex molecular processes (48), practically summarizable into two main mechanisms:

### 5.1. Melatonin enhances antioxidant agents

ANT increases oxidative stress by generating ROS via a redox cycling mechanism. The complex I of electron transport chain reduces DOX, a quinone compound, to a semiquinone which in turn donates an unstable electron to an oxygen molecule and formed a dangerous ROS, namely superoxide anion (O2 ^−^) (52). Normally, antioxidative enzymes defend against oxidative damaged via the detoxification of the generated ROS. In particular, the circulant antioxidant agent SOD is able to convert the superoxide anion (O2 ^−^) to hydrogen peroxide (H2O2), which is then antioxidized by catalase and glutathione peroxidase enzyme into an harmless water molecule (53). ANT decrease the levels and activity of SOD (13). The present meta-analysis highlights that in mouse models exposed to ANT, the use of melatonin significantly increases blood levels of the antioxidant agent SOD compared to controls without melatonin (Figure 4) (51, 54).

GSH, the most plentiful thiol-containing substance of low molecular weight in cells, is a crucial antioxidant and antidote in all mammalian tissues, particularly in the cardiomyocytes. Indeed, heart GSH quenches oxidizing substances (such as reactive hydroxyl free radicals, peroxynitrite, and H2O2) directly or reduces H2O2 to water (55). In this instance as well, melatonin treatment appears capable of restoring and maintaining significantly higher levels of heart GSH in mouse models exposed to ANT (Figure 3) (10, 22). Finally, melatonin also appears to exert antioxidative properties via direct scavenging of ROS, mainly by single electron transfer, hydrogen transfer, and radical adduction formation (46).

### 5.2. Melatonin directly mitigates lipid peroxidation, a product of oxidative damage

One of the consequences of uncontrolled oxidative stress (imbalance between the prooxidant and antioxidant levels in favor of prooxidants) is cells, tissues, and organs injury caused by oxidative damage. In particular, it has long been recognized that high levels of ROS can inflict direct damage to lipids (56). Indeed, ROS attack lipids containing carbon-carbon double bond(s), especially polyunsaturated fatty acids (PUFAs), with oxygen insertion resulting in lipid peroxidation process whose main secondary product is MDA (57). Under high lipid peroxidation rates the extent of oxidative damage overwhelms repair capacity and the cells auto-induce apoptosis or necrosis programmed cell death (58). In this setting, MDA has been widely used for many years and it is currently still adopted as a convenient biomarker for evaluation of lipid peroxidation and, in general, of oxidative stress (59, 60). In mouse models ANT significantly increase the level of lipid peroxidation products, especially MDA, but also 4-hydroxyalkenals, thiobarbituric acid reactive substances, conjugated dienes and protein carbonyl (32, 19). Extensive basic science literature has suggested that the use of melatonin can directly mitigate lipid peroxidation induced by ANT, mainly assessed by detecting MDA levels ​​in the heart of mouse models (30, 28). Supporting this hypothesis, our meta-analysis results indicate that mice exposed to ANT and administered melatonin exhibit significantly lower MDA (i.e., lipid peroxidation) levels compared to controls (Figure 2).

Although not the primary focus of this systematic review, there are additional mechanisms beyond the discussed protection against oxidative damage that contribute to the cardioprotective effect of melatonin against ANT-induced cardiotoxicity (48). Notably, studies conducted both *in vitro* and *in vivo* (using mouse models) suggest that melatonin exerts its cardioprotective effects by safeguarding, regenerating, and enhancing mitochondrial functions (61). Indeed, as it is known, ANT rapidly disrupts the structural and functional integrity of mitochondria, resulting in advanced mitochondrial dysfunction (62). Melatonin-induced normalization of Sirt1/Nrf2 pathways increases the levels of PGC-1*α* and ultimately suppresses the expression of Drp-1, leading to a reduction in mitochondrial fission. Moreover, melatonin supports mitochondrial biogenesis by preventing the decrease of PGC-1*α* and normalizing signal transmission in models of ANT-induced cardiotoxicity (16). These protective effects counteract mitochondrial dysfunction and improve mitochondrial clearance, also providing essential antiapoptotic actions (19). Melatonin also regulates ANT-induced myocardial cell death through a direct impact on the essential autophagy and mitophagy pathways (63). Due to these melatonin's antiapoptotic effects, there might be concerns about its potential interference with the effectiveness of ANT cancer treatment. Fortunately, evidence suggests that melatonin has dual effects on apoptosis, selectively enhancing the antiapoptotic effect on normal cells while triggering apoptotic pathways in cancer cells. Therefore, it does not appear to compromise the efficacy of chemotherapy (64).

Moreover, melatonin stabilizes iron metabolism (65), preventing excessive formation of the Fenton reaction and ferroptosis, which play a significant role in the development of ANT-induced cardiotoxicity (66).

In addition, ANT induces biochemical stress in the myocardium, leading to a switch in metabolic substrate utilization from fatty acids to glucose. This metabolic switch leads to fatty acids accumulation, which can result in ROS formation, with subsequent mitochondrial dysfunction and endoplasmic reticulum stress-related protein misfolding. Melatonin and its active metabolites appear to limit and decelerate all the above-mentioned changes (67).

Finally, melatonin exhibits significant anti-fibrotic potential achieved by inhibiting the lncR-MALAT1/miR-141-mediated activation of NLRP3 inflammasome and TGF-*β*1/Smad signalling pathways stabilization (68). This effect is particularly relevant concerning the pro-fibrotic effects of ANT, as the myocardium is a highly differentiated morphological unit, and in response to injury and cell death, fibroblast differentiation with subsequent production of connective tissue and fibrosis occurs (69).

Finally, the ability of melatonin to protect the heart from the ANT-induced oxidative damage, together with the additional cardioprotective mechanisms discussed above, would seem to directly impact cardiomyocyte viability. As supported by the results of our meta-analysis, melatonin is associated with significantly lower levels of myocardionecrosis indexes (*i.e.*, CK-MB) than controls in mice exposed to toxic doses of ANT (Figure 5). The protective effect on the myocardium could have significant clinical implications, suggesting that melatonin could mitigate ANT-induced myocardial damage and consequently prevent the development of left ventricular dysfunction and other manifestations of cardiotoxicity. In support of this hypothesis, some studies, conducted on murine models, have shown that melatonin ameliorates the reduction of left ventricular ejection fraction, of stroke volume, and of fractional shortening that are induced by ANT (70, 71). Indeed, encouraging echocardiographic findings from Liu et al. propose that melatonin may alleviate ANT-induced systolic dysfunction, as assessed by left ventricular ejection fraction and left ventricular fractional shortening (16). To date, there is only a single human study that has evaluated the impact of melatonin in patients with cancer. This study was a randomized trial involving 250 individuals with advanced-stage solid tumors and compromised functional status, some of them treated with ANT. The findings of this study were encouraging, indicating that melatonin potentially enhanced the 1-year survival rate and decreased the occurrence of cardiotoxicity (33).

## 6. Conclusions

The findings of the present comprehensive systematic revision of the basic science literature, along with the conducted meta-analysis, support the hypothesis that melatonin mitigates the oxidative damage caused by ANT and the subsequent myocardial damage. Consequently, it holds promise as a potential effective clinical approach for preventing and/or treating ANT-induced cardiotoxicity in humans. However, high quality clinical studies are thus warranted, and indeed to be recommended to further evaluate the use of melatonin as a preventative/treatment strategy for ANT-induced cardiotoxicity.

## Data Availability

The data analyzed in this study is subject to the following licenses/restrictions: Created dataset is available upon request. Requests to access these datasets should be directed to andreafaggiano95@gmail.com.

## Supplementary material

Supplementary Figure S1 Funnel plot assessing publication bias for standard means difference (SMD) of Superoxide Dismutase (SOD) in melatonin treated mice and controls. Observed (white symbols) and imputed (black symbols) values.
