# Supplementary figures and images for: Melatonin mitigates oxidative damage induced by anthracycline: a systematic-review and meta-analysis of murine models
Source: Front Cardiovasc Med. 2023 Nov 23;10:1289384. doi: 10.3389/fcvm.2023.1289384 (PMC10701532; doi:10.3389/fcvm.2023.1289384)

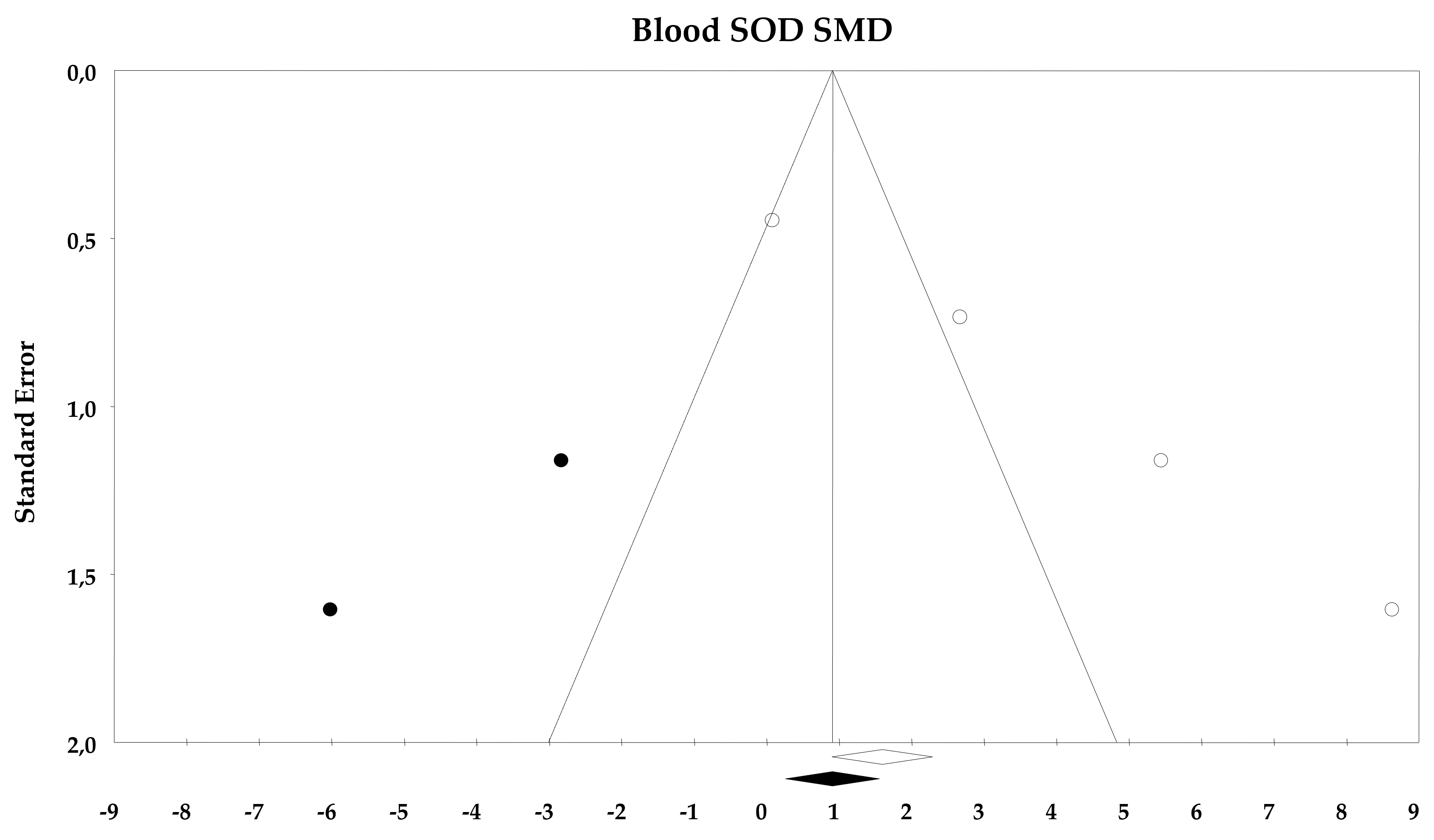

Supplement: Supplementary Figure S1 — Funnel plot assessing publication bias for standard means difference (SMD) of Superoxide Dismutase (SOD) in melatonin treated mice and controls. Observed (white symbols) and imputed (black symbols) values. [file Image1.tif]
